# Editorial Comment: Can extended upper pole ureterectomy prevent ureteral stump syndrome after proximal approach for duplex kidneys?

**DOI:** 10.1590/S1677-5538.IBJU.2020.0686.1

**Published:** 2021-07-20

**Authors:** José Murillo Bastos

**Affiliations:** 1 Universidade Federal de Juiz de Fora Hospital Universitário Divisão de Urologia Pediatrica Juiz de ForaMG Brasil Divisão de Urologia Pediatrica, Hospital Universitário da Universidade Federal de Juiz de Fora (UFJF), Juiz de Fora, MG, Brasil; 2 Faculdade de Ciência Médicas e da Saúde de Juiz de Fora Hospital e Maternidade Therezinha de Jesus Juiz de ForaMG Brasil Hospital e Maternidade Therezinha de Jesus da Faculdade de Ciência Médicas e da Saúde de Juiz de Fora (SUPREMA), Juiz de Fora, MG, Brasil

## COMMENT

I would like to congratulate the authors for this interesting study that adds some new data to the discussion on whether we should or should not extend our ureteral dissection when performing heminephrectomy in children. The majority of the studies discussing this matter are about a decade old or more and most of them including a fairly small group of patients.

Previous reports have shown ureteral stump complications to be as low as 12% (1, 2) and the authors advocate that a distal ureteral stump can be left safely, even in children that vesicoureteral reflux is the indication for nephrectomy. In these series, the most common complication was recurrent urinary tract infections that resolved after ureteral stump removal. In another study, Caluwe et al. also left residual ureteral stumps and 20% of the patients in their series needed a secondary procedure to treat complications of the residual stump (3).

One of the reasons to leave the distal portion of the ureter, especially in duplex system, is the common sheath of the upper and lower pole ureters, which makes the dissection more complicated, exposing the healthy unit ureter to devascularization and surgical lesions. Another reason is a second groin incision to extend the ureteral dissection toward its distal portion in those undergoing open surgery, which increases surgical morbidity. On the other hand, laparoscopic surgery can reduce morbidity of a second incision, but a careful dissection of the lower ureteral portion is still necessary.

On the contrary, Cezarino et al. have shown a 30% complication rate associated with partial ureteral removal compared to 6% in those submitted to extended ureteral excision, but they were not able to find any factors associated with these complications (4). Recently, Escolino et al., 2016, have shown a higher incidence of complications in those children presenting vesicoureteral reflux when a longer ureteral stump was left in place and recommend the total excision of the ureter in these patients (5).

Although there will always be a debate on total versus partial ureteral excision, one should carefully discuss with the family the pros and contras of doing a total ureteral excision and the chances of needing a secondary surgery. In my opinion, if a laparoscopic approach is used, a careful dissection and removal of the whole ureter should be performed to avoid such complications and extra surgical procedures, that can occur in up to 30% of the cases, as shown by Cezarino et al., herein (4). In cases of an open surgery, the need for an extra groin incision should be taken in consideration and left for a later moment, as a secondary procedure, if ureteral stump syndrome occurs.
